# Deep learning for improving non-destructive grain mapping in 3D

**DOI:** 10.1107/S2052252521005480

**Published:** 2021-07-15

**Authors:** H. Fang, E. Hovad, Y. Zhang, L. K. H. Clemmensen, B. Kjaer Ersbøll, D. Juul Jensen

**Affiliations:** a Department of Mechanical Engineering, Technical University of Denmark, Kgs. Lyngby 2800, Denmark; b Department of Applied Mathematics and Computer Science, Technical University of Denmark, Kgs. Lyngby 2800, Denmark

**Keywords:** grain mapping, X-ray diffraction, tomography, deep learning, computer vision, background noise, spot segmentation, LabDCT

## Abstract

Laboratory X-ray diffraction contrast tomography (LabDCT) is a novel imaging technique for non-destructive 3D characterization of grain structures. An accurate grain reconstruction critically relies on precise segmentation of diffraction spots in the LabDCT images. The conventional method utilizing various filters generally satisfies segmentation of sharp spots in the images, thereby serving as a standard routine, but it also very often leads to over or under segmentation of spots, especially those with low signal-to-noise ratios and/or small sizes. The standard routine also requires a fine tuning of the filtering parameters. To overcome these challenges, a deep learning neural network is presented to efficiently and accurately clean the background noise, thereby easing the spot segmentation. The deep learning network is first trained with input images, synthesized using a forward simulation model for LabDCT in combination with a generic approach to extract features of experimental backgrounds. Then, the network is applied to remove the background noise from experimental images measured under different geometrical conditions for different samples. Comparisons of both processed images and grain reconstructions show that the deep learning method outperforms the standard routine, demonstrating significantly better grain mapping.

## Abstract 2

A deep learning neural network has been developed to efficiently and accurately clean the background noise in experimental lab-based X-ray diffraction images. A better spot segmentation is obtained and thus grain mapping in 3D is improved compared with the conventional method.

## 1. Introduction   

Non-destructive 3D characterization of grain structures (sizes, shapes and orientations) is indispensable in understanding the microstructural evolution in the bulk of polycrystalline materials. Such techniques are well established at large synchrotron X-ray facilities and provide grain mapping at length scales from tens of nanometres to millimetres, and temporal resolution from microseconds to hours (*e.g.* Larson *et al.*, 2002 ▸; Poulsen, 2004 ▸, 2020 ▸; Suter *et al.*, 2006 ▸; Ludwig *et al.*, 2008 ▸; Simons *et al.*, 2015 ▸). To broaden the use of non-destructive grain mapping by offering such possibilities at home laboratories, laboratory X-ray diffraction contrast tomography (LabDCT) has been developed (King *et al.*, 2013 ▸, 2014 ▸; McDonald *et al.*, 2015 ▸). This novel technique has already been demonstrated to be very useful in 3D/4D studies of metals and alloys (McDonald *et al.*, 2017 ▸; Sun *et al.*, 2019 ▸, 2020 ▸; Lei *et al.*, 2021 ▸).

A 2D detector is typically placed behind the sample to record diffraction spots in a transmission geometry in LabDCT. Grain microstructures are reconstructed from the spots collected at different sample rotations. As the spots often suffer from undesired background noise, precise spot identification may be difficult, which in turn leads to mistakes in the grain reconstructions. Conventionally, rolling median processing is used for reducing the noise followed by various filters to enhance the spot contrast. This approach works fine for spots with good signal-to-noise ratios (SNRs) but it can lead to over or under segmentation of spots with low SNRs. As the background intensities vary over projections and time due to variations of X-ray source and sample thickness, the background noise cannot always be removed completely by the rolling median process, resulting in poor spot segmentation. Furthermore, it requires extensive human expertise to tune and optimize the processing parameters.

In recent years, machine-learning methods have been adopted by materials scientists to successfully identify or restore features of interest from microscopic or tomographic images (*e.g.* Larmuseau *et al.*, 2021 ▸; Jiang *et al.*, 2020 ▸; DeCost *et al.*, 2017 ▸; Dimiduk *et al.*, 2018 ▸). Particular interest has also increased within X-ray diffraction imaging. For example, a machine-learning method was developed to locate Bragg peaks in high-energy X-ray diffraction images at a much faster speed than conventional pseudo-Voigt fitting (Liu *et al.*, 2021 ▸). A deep learning (DL) model trained with synthetic data was established to reconstruct a single-particle image in Bragg coherent diffraction imaging (Wu *et al.*, 2021 ▸). We also established a DL model for processing LabDCT images (Hovad *et al.*, 2020 ▸). In that work, the model was trained with synthetic data containing very simple (and actually not realistic) noise and still turned out to be quite efficient in removing background noise for LabDCT images.

Encouraged by the first attempt, in this work we develop a new DL model by synthesizing images containing fully realistic noise. This is carried out by establishing a flexible method to generate input and ground-truth images using a forward simulation model combined with a generic approach to extract features of the experimental background noise. The features of the synthesized images are carefully tuned to capture the main aspects of the experimental images. The trained model turns out to be excellent in removing the background noise as confirmed by applying this model to real unseen experimental images. Comparisons of spot-segmented images and complete grain reconstructions both show that the DL method outperforms the standard routine.

## 2. Data and methods   

### 2.1. Data for the DL networks   

To avoid intensive labor manual training of the DL network, a forward simulation model was used to generate LabDCT projections. Each projection contains diffraction spots from the first four {*hkl*} families of all grains in artificially created samples, a constant background and a central region with zero pixel values to mimic the beam stop shielding the direct beam. To maximize the variety of the spot features such as sizes and intensities, we synthesized three cylindrical aluminium samples with different grain size distributions covering the most common grain size ranges for LabDCT studies. Table 1 ▸ shows the main characteristics of the input samples. The grain orientations were generated randomly and no misorientation was present within the grains, *i.e.* zero mosaicity.

**Figure 1:**
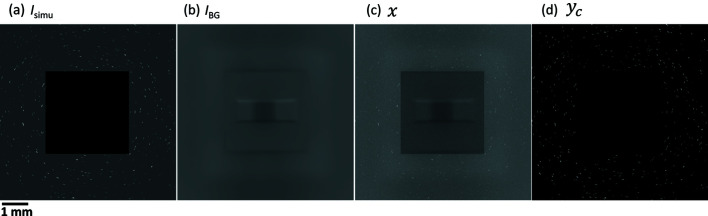
Data for training the DL network. (*a*) A simulated LabDCT image (*I*_simu_) containing spots, a constant background and a central black area (pixel value = 0) from the sample D35. (*b*) A normalized background-noise image (*I*_BG_, gray values between 0 and 1) containing only long-range noise extracted from an experimental dataset. (*c*) An input image (*x*) containing both spots and noise. (*d*) A content image (*y*_c_) containing spots only.

The grains were meshed into polyhedrons and the diffracted intensity of each lattice plane of each polyhedron was computed; for further details see Fang *et al.* (2020 ▸). Diffraction spots were thus generated by summing all the diffraction intensities for each pixel on a virtual detector (2032 × 2032 pixels with an effective pixel size of 3.36 µm). The simulations were performed in a Laue focusing geometry: the sample-to-source distance (*L*_ss_) was the same as the sample-to-detector distance (*L*_sd_) and equal to 11 mm. An X-ray spectrum from a tungsten anode X-ray tube operating at 140 kV was used as the photon source. For each sample, 181 projections were simulated in 2° steps for a 360° rotation. Fig. 1 ▸(*a*) shows an example of the simulated LabDCT projection (*I*_simu_). The forward simulation model can handle any sample geometry, any grain size and any X-ray spectrum but here we only performed simulations for the most common experimental circumstances. More details of the forward simulation model can be found in the work of Fang *et al.* (2020 ▸) and the code is available at https://github.com/haixingfang/LabDCT-forward-simu-model.

The input image (*x*) for the DL networks was created by combining the simulated LabDCT image with an image of the background noise. The latter image was produced by extracting and normalizing the background noise from an experimental LabDCT dataset. A generic method was established to extract background noise from any type of experimental images. An example of the normalized background-noise image (*I*_BG_) is shown in Fig. 1 ▸(*b*). Here, the background-noise image mainly contains the long range (low frequency) noise that depends on the sample, values of *L*_ss_ and *L*_sd_, the X-ray beam size, as well as the intensity. To also mimic the short range (high frequency) noise, Poisson-type noises were subsequently added. For details on this method, see Appendix *A* [App appa].

The arithmetic operation to create the input image *x* in a pixel-by-pixel manner can be expressed as
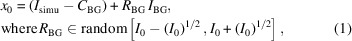
and
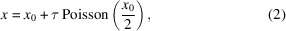
where *x*_0_ is the image containing the spots and the long-range noise, *C*_BG_ is the background constant value of *I*_simu_, *R*_BG_ is a value randomly generated in the range [*I*_0_ − (*I*_0_)^1/2^, *I*_0_ + (*I*_0_)^1/2^] determined by a constant *I*_0_, Poisson(*x*_0_/2) represents the short-range noise following a Poisson distribution with a mean value of *x*_0_/2 for each pixel, and τ is a factor to tune the noise level. In this work we set τ = 1 and *I*_0_ = 110, which is a common background-pixel value for experimental LabDCT images (Lindkvist *et al.*, 2021 ▸). Since *R*_BG_ is a random number and the Poisson noise is added, variances are retained for each input image (*x*) even when the same combination of *I*_simu_ and *I*_BG_ are used. This matches the dynamic changes of the X-ray beam characteristics in real experiments. An example of the input image is shown in Fig. 1 ▸(*c*) ▸. A plot of the signal-to-noise distribution is presented in Fig. S1(*a*) of the supporting information.

Fig. 1(*d*) ▸ shows a content image (*y*_c_) that serves as the ground truth for the DL network, corresponding to what would have been identified in a perfect manual training of the network. The spots in the content images were segmented from the simulated images *I*_simu_ using a Laplacian of Gaussian (LoG) approach (more details on this approach are described in Section 2.4). Since the background is constant, this segmentation precisely retains the spot shapes. In *y*_c_ the pixel values of diffraction spots are 255 and the remaining pixels, *i.e.* the background, have a value of 0. In total, we synthesized 1086 images in an eight-bit format for each *x* and *y*_c_ from a random combination of three forward simulated image datasets and three different types of *I*_BG_ extracted from real experimental images.

### 2.2. DL algorithm   

**Figure 2:**
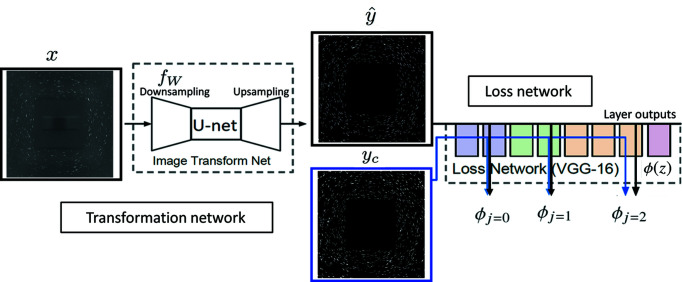
Deep learning neural networks consisting of a transformation network and a loss network. Adapted from the works of Johnson *et al.* (2016 ▸) and Hovad *et al.* (2020 ▸).

As shown in Fig. 2 ▸, the DL method consists of two components: an image transformation network *f*_*W*_ and a loss network ϕ(*z*), where 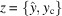 (Johnson *et al.*, 2016 ▸). The image transformation network transforms input images *x* into output images 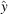 via mapping 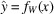 with a deep convolutional neural network parameterized by weights *W*. In this work, the transformation network has an architecture similar to the original U-Net (Ronneberger *et al.*, 2015 ▸; Çiçek *et al.*, 2016 ▸), but is featured differently by having a pre-trained model as the backbone. This network is referred to as the ‘dynamic U-Net’ network. Here, ResNet34 (https://www.kaggle.com/pytorch/resnet34) is used as the pre-trained model.

There are in total 54 convolutional layers in the dynamic U-Net network. This network consists of a contracting path (image size is decreasing, downsampling) and an expansive path (image size is increasing, upsampling). The contracting path follows the typical architecture of a convolutional network. It consists of the repeated application of convolutions, each typically followed by a batch normalization, a rectified linear unit (ReLU) or a max-pooling operation for downsampling. The number of feature channels is doubled at each downsampling step. Every step in the expansive path consists of an upsampling of the feature map followed by a convolution that halves the number of feature channels, a concatenation with the correspondingly cropped feature map from the contracting path, and several convolutions, each typically followed by an ReLU. To avoid checkerboard artifacts for upsampling images, a sub-pixel convolutional neural network, PixelShuffle (Shi *et al.*, 2016 ▸), is used. Batch normalization is also used in several places in the upsampling to control the gradients during training. Average 2D pooling is used to get the dimensions right with respect to odd numbers of image size. At the final layer a convolution is used for mapping each 64-component feature vector to the given output image size. More details on the layers in the dynamic U-Net are summarized in Table S1 of the supporting information.

The loss network defines loss functions that measure the difference between the output image 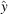 and a target content image *y*_c_ at different layers. In this work, we use a 16-layer VGG network (Simonyan & Zisserman, 2015 ▸) and compute three outputs (*j* = 0, 1, 2) for the loss functions. Instead of only encouraging each pixel of the output image 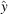 to exactly match that of the content image *y*_c_, the output image is also encouraged to have similar features as computed by the loss network. Therefore, three contributions to the total loss, 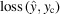, are considered: pixel loss, feature loss and Gram loss. The pixel loss is the normalized Euclidean distance between 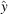 and *y*_c_. The feature loss is the normalized Euclidean distance between feature maps of 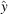 and *y*_c_ at the *j*th output from the loss network, denoted as 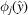 and ϕ_*j*_(*y*_c_), respectively. The Gram loss is the squared Frobenius norm of the difference between the Gram matrices of 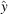 and *y*_c_ at the *j*th output, denoted as 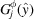 and 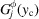, respectively. The total loss can thus be expressed as
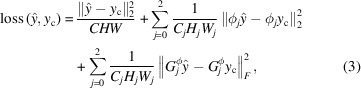
where *C* is the number of image channels, and *H* and *W* are image height and width in pixels, respectively. More details on the DL networks used here can be found elsewhere (Johnson *et al.*, 2016 ▸; Hovad *et al.*, 2020 ▸).

### 2.3. Model implementation   

The DL model was implemented using a Jupyter notebook with Python codes based on the fast.ai library (https://github.com/fastai/course-v3/blob/master/nbs/dl1/lesson7-superres.ipynb). The 1086 generated images for both *x* and *y*_c_ were resized using bilinear interpolations to *H* × *W* = 600 × 600, and split into a training dataset (85% = 924 images) and a validation dataset (15% = 162 images). Notably, the resizing reduces the image resolution and may cause an aliasing effect on the spot shapes. However, this effect is limited to one to two pixels on the spot edges only and thus does not significantly affect the DL performance. The DL model was first trained with a fixed learning rate (lr = 0.01) and then with the learning rate unfrozen. A batch size of three was used for training the model. During each epoch of training, training loss (loss value for the training dataset) and validation loss (loss value for the validation dataset) were computed. The model was trained for a maximum of 50 epochs where it was observed that the validation loss stopped decreasing. The training took ∼40 h using an NVIDIA Quadro P5000 with a memory size of 16 GB. After the model was trained, it took only 40 s to remove the background noise and identify the spots in a typical experimental LabDCT dataset consisting of 181 images. The final DL output images have a size of 1200 × 1200, which were subsequently used for grain reconstruction. The source programming codes, training dataset, trained model and DL output images will be published on Materials Commons and will be publicly accessible (https://doi.org/10.13011/m3-2z1n-qh56).

### 2.4. Experimental LabDCT measurements and grain reconstructions   

With the aim of quantifying how efficient the trained DL network is to identify spots in real images and how this new procedure compares with the standard routine, a fully recrystallized pure iron (99.99 wt% Fe) sample was characterized with a commercial LabDCT instrument (Zeiss Xradia 520 Versa X-ray microscope). Scans were performed in a Laue focusing geometry, *i.e.* *L*_ss_ = *L*_sd_ = 13.0 mm. This distance is slightly different from that used in the training. The reason for choosing a different distance is to further challenge the DL network. Diffraction signals were recorded by a detector (2032 × 2032 pixels) with an effective pixel size of 3.36 µm. A beam stop with an area of 2.5 × 2.5 mm was placed between the sample and the detector to block the direct transmitted beam. A total of 181 diffraction images with an exposure time of 500 s were acquired during a full sample rotation of 360°, followed by recording 1601 absorption tomographic projections (exposure time of 1.5 s) for reconstructing the sample volume. An accelerating voltage of 160 kV and a power of 10 W were used for all the scans. The LabDCT projections were then processed with the standard routine and the DL method.

Using the standard routine, the LabDCT projections were first processed by a rolling median correction through the image stack to remove most of the background noise with the beam-stop area excluded for further analysis. The diffraction spots were then segmented using an LoG based approach. This approach includes four parameters: a background value, a standard deviation (SD) for the Gaussian filter, a percentage value and a minimum spot size, which are required to be carefully tuned for optimizing spot segmentation. In this approach the rolling-median-corrected image is subtracted by the background value, then smoothed with the Gaussian filter (the SD must be assigned) and subsequently processed with a Laplacian operator. All the connected components in the resulting image are then segmented one-by-one by applying a threshold value that is a certain percentage of the maximum intensity of the connected component. At last, any identified spots smaller than a certain size are removed because they are likely to be noise. More details on the standard routine for spot segmentation can be found elsewhere (Fang *et al.*, 2021 ▸).

The trained DL networks were directly applied to the LabDCT projections, removing the background noise without the need to tune any parameter. Since all the background noise is cleaned (gray values become zero) and only the spots are left by this method, segmentation to create binary images is straightforward.

Based on the binary images output from either the standard routine or the DL method, grain structures with a voxel size of 2.5 µm were reconstructed using a commercial software (*GrainMapper3D* version 2.3, developed by Xnovo Technology ApS). A standard set of parameters for the grain reconstruction (Fang *et al.*, 2021 ▸) were used and held fixed for all reconstructions. Therefore, the effects of reconstruction parameters were excluded and the reconstruction is only dependent on the method for creating the binary images. Detailed descriptions of *GrainMapper3D* can be found in the works of Bachmann *et al.* (2019 ▸) and Oddershede *et al.* (2019 ▸).

### 2.5. Comparison of the reconstructed grain structures obtained by LabDCT with the ground truth obtained by synchrotron measurements   

The two LabDCT reconstructed grain structures, based on the standard routine and the novel DL method, were compared with that obtained by synchrotron DCT (SR-DCT) measurements of the same iron sample. As reported in previous studies (Reischig *et al.*, 2013 ▸; Renversade *et al.*, 2016 ▸; Johnson *et al.*, 2008 ▸; Syha *et al.*, 2013 ▸), SR-DCT has an orientation resolution of <0.1°, a detection limit of ∼5 µm (smallest detectable grain in terms of equivalent spherical diameter) and a spatial resolution [accuracy of grain boundary (GB) position] of ∼1.5 µm for fully recrystallized grain structures. LabDCT has a similar orientation resolution (∼0.1°), but a minimum detectable grain size of the order of >20–40 µm (Bachmann *et al.*, 2019 ▸) and a spatial resolution of 4.4–7 µm for grains larger than 40 µm (McDonald *et al.*, 2015 ▸, 2021 ▸), whilst the spatial resolution becomes worse for smaller grains (Fang *et al.*, 2021 ▸). Although the indexing of small grains might not be fully correct even with SR-DCT, the spatial resolution and detection limit for SR-DCT are both much better than LabDCT. Therefore, the grain structure obtained by SR-DCT is used as the ground truth in the present analysis. This grain structure dataset is referred to as SR-DCT. Details of the SR-DCT measurement are reported by Zhang *et al.* (2018 ▸), and information about the DCT setup and data processing can be found in the works of Johnson *et al.* (2008 ▸) and Ludwig *et al.* (2008 ▸, 2009 ▸).

Grain indexing was compared between each LabDCT reconstruction and the ground truth. GB positions for each commonly indexed grain were also compared and the differences were quantified. The GB deviation (ɛ_GB_) for each voxel in the SR-DCT dataset was calculated as the Euclidean distance between this voxel and the nearest voxel on the boundary of the paired grain in the LabDCT dataset. The average GB deviation (δ_GB_) can thus be calculated as 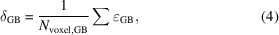
where *N*_voxel, GB_ is the total number of GB voxels in the SR-DCT dataset. Details of the method for such comparisons can be found in the work of Fang *et al.* (2021 ▸). By comparing the grain indexing and the GB positions found in the LabDCT datasets with the ground truth, we can identify whether the DL method can outperform the standard routine for the grain mapping or not.

## 3. Results   

### 3.1. DL training   

**Figure 3:**
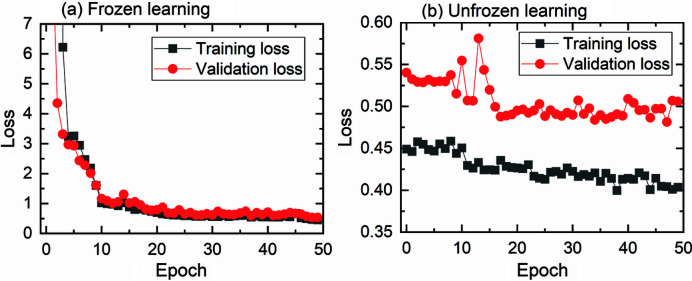
Training and validation loss as a function of epoch number during (*a*) frozen learning with a constant learning rate of 10^−2^ and (*b*) unfrozen learning.

The aim of the training of the DL network is to minimize the validation loss. Fig. 3 ▸ shows the training loss and validation loss as a function of training epoch. During the first step with frozen learning, the loss values rapidly decrease in the first ten epochs and then slowly decrease further [Fig. 3 ▸(*a*)]. During the subsequent step with unfrozen learning, the training loss continues to decrease while the validation loss becomes steady after 15 epochs [Fig. 3 ▸(*b*)]. Therefore, the training was stopped at the 49th epoch. As the parameter optimizing of the DL networks is complex during training, the loss curve is often not as smooth as the validation-loss curve in Fig. 3 ▸(*b*).

After the training is complete, the noise removal is found to be very efficient. An example of the noise removal for the input image *x* from the validation dataset can be seen in Fig. 2 ▸. The figure shows that the DL output image 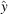 contains only spots and no background noise, *i.e.* very similar to the content image *y*_c_.

### 3.2. Performance of the DL model for background removal in real experimental images   

**Figure 4:**
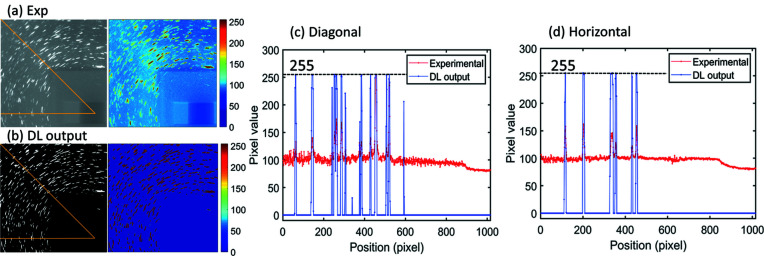
DL output for an experimental LabDCT image. (*a*) The experimental image (with pixel values between 38 and 255), (*b*) the DL output image (with pixel values between 0 and 255, only 1/4 of the full images are shown here), and pixel-value profiles of line scans in (*c*) diagonal and (*d*) horizontal directions of the two images. The dashed lines in (*c*) and (*d*) indicate the maximum pixel value of 255.

**Figure 5:**
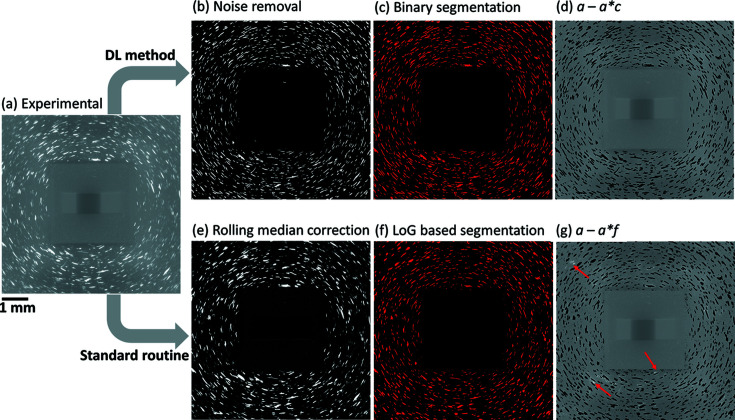
Comparison of the steps to create binarized spot images from (*a*) an experimental image using (*b*), (*c*) the DL method and (*e*), (*f*) the standard routine. Furthermore, (*d*) and (*g*) are the results of arithmetic operations on the experimental and binary images: *a* − *a* *c* and *a* − *a* *f*, respectively, showing the performance of the segmentations. Red arrows in (*g*) mark spots that are not segmented by the standard routine.

After being trained, the DL model was applied to process the experimental LabDCT images. An example of the DL output and the experimental input image is shown in Figs. 4 ▸(*a*) and 4 ▸(*b*) (here only one quarter of the image is shown – the whole image is shown in Fig. 5). It can be seen that the background is totally black, leaving only diffraction spots brightly visible in the DL output. A quantitative comparison is shown by pixel-value profiles along two line scans: diagonal as well as horizontal [Figs. 4 ▸(*c*) and 4 ▸(*d*), respectively]. Both line scans show that the spots are recovered with sharp peaks and the background intensities are reduced to zero everywhere, regardless of location (such as the corner and the beam-stop region) in the image. This confirms that the background noise has been completely removed. The intensities of most spots are recovered to 255 (the maximum pixel value for an eight-bit image). A few spots, recovered from very weak experimental spots, have lower intensities after the DL cleaning [*e.g.* the small peak for the diagonal line scan shown in Fig. 4 ▸(*c*)]. This is reasonable because the model has not been trained to exactly restore all the pixel values, as indicated by the non-zero loss value at the end of the training [see Fig. 3 ▸(*b*)], although a minimal loss value has been reached. Nevertheless, Fig. 4 ▸ shows that all the recovered spots have intensities significantly higher than zero. This means that the spots in the output image can be easily and precisely segmented by applying a single threshold value.

A comparison of the segmented binary images obtained by the DL method and the standard routine is shown in Fig. 5 ▸. Using the DL method with the experimental image as the input, the output only contains spots [see Fig. 5 ▸(*b*)], and thus it is straightforward to segment (here with a threshold value of five) and create the binary image as shown in Fig. 5 ▸(*c*). Using the standard routine, a rolling median correction was first applied to reduce the background noise of the experimental image [resulting in Fig. 5 ▸(*e*)], and then an LoG-based approach was employed for spot segmentation. Given an optimized combination of these parameters, the segmented image is shown in Fig. 5 ▸(*f*). We also plotted the distribution of the SNRs in comparison with the synthesized DL input images [see Fig. S1(*b*)].

From the images showing the difference between the segmentation and the experimental image [Figs. 5 ▸(*d*) and 5 ▸(*g*)], we can see that spots are well segmented using the DL method [no visible bright areas are seen in Fig. 5 ▸(*d*), such bright areas would mean bad spot segmentation]. Using the standard procedure, there are some bright areas corresponding to mis-segmented spots or parts of spots, which are still clearly seen in Fig. 5 ▸(*g*) (marked by the arrows). Upon close examination it is found that these bright areas very often are connected to spots with higher intensities. There may be two reasons for this mis-segmentation by the standard procedure: (i) during the rolling median correction, parts of spots can be removed when too many spots at different projections are present at the same pixel position through the image stack, and (ii) during LoG processing the threshold value determined by the percentage of maximum intensity of the whole connected region is too high to segment the weaker spots.

Deficiencies of the standard routine thus demonstrate two clear benefits gained by the DL model: a more precise spot segmentation and no need to tune parameters.

### 3.3. Comparison of reconstructed grain structures   

#### 3.3.1. Grain indexing   

**Figure 6:**
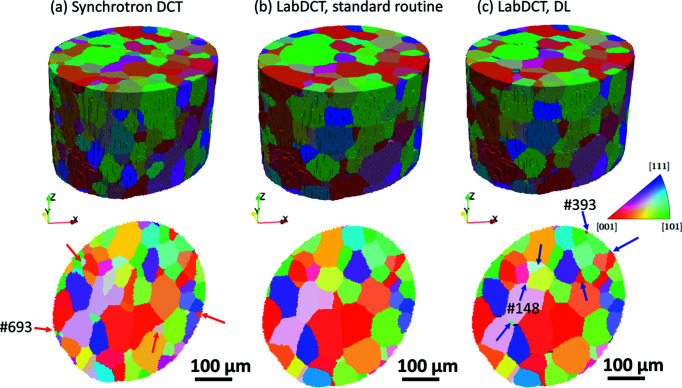
Grain structures visualized in 3D in the top row and as slices normal to the *Z* axis (sampled at a distance of 115 µm from the top surface) in the bottom row. Grains are colored with *Z*-direction inverse-pole-figure colors. (*a*) SR-DCT, and LabDCT datasets obtained using (*b*) the standard routine and (*c*) DL. Grains of interest are marked by red and blue arrows in the slices (some are shown with their grain IDs).

Fig. 6 ▸ shows the LabDCT reconstructions by the standard routine (Lab-Routine) and by DL (Lab-DL), together with the synchrotron dataset (SR-DCT), the latter being the ground truth. While the grain structures are largely consistent, significant differences in grain indexing and grain shapes can be seen upon close examination, especially from 2D slices. Some grains are seen in the slice of SR-DCT but are not seen in any of the two LabDCT slices [see the red arrows in Fig. 6 ▸(*a*)]. By checking slices at other locations, we found that all these grains except grain #693 exist in other sections of both LabDCT datasets, indicating a significant spatial shift for reconstructions of these grains. Comparison of the LabDCT dataset with SR-DCT shows that more grains are present in the same slice of Lab-DL than Lab-Routine, as seen for the grains marked by the blue arrows in Fig. 6 ▸(*c*). Among these grains, grain #148 and #393 cannot be found in other slices of Lab-Routine, which means that they are not indexed by Lab-Routine.

Statistics of the grain indexing are given in Table 2 ▸. Both the two LabDCT datasets index far fewer grains (mostly small ones of <20 µm) than SR-DCT, resulting in a slightly larger value of the average grain size 〈*D*〉. Interestingly, the 〈*D*〉 value in Lab-Routine is closer to that of SR-DCT than Lab-DL. This seems to imply that Lab-Routine provides a better grain reconstruction. However, comparisons of the grain volumes indicate that the volumes of small grains are more severely underestimated (which is less accurate) in Lab-Routine than in Lab-DL, leading to a slightly smaller average grain size in Lab-Routine, although there are more grains indexed in Lab-DL (see the plot of grain size distributions in Fig. S2 and the total volume of the reconstructed grains in Table S2).

All the grains in the datasets can be classified into four groups: one-to-one indexed, one-to-multi indexed (one grain in SR-DCT is indexed by more than one grain in the LabDCT dataset), false-negatively indexed (a grain that is not indexed in the LabDCT dataset but actually exists in the ground truth) and false-positively indexed (a grain that is indexed in the LabDCT dataset but actually does not exist in the ground truth). The first two groups are true-positively indexed. It is found that there are 12 more one-to-one indexed (mostly grains of <30 µm) and 11 fewer false-negatively indexed grains in Lab-DL than Lab-Routine, whilst the number of one-to-multi and false-positively indexed grains is very similar. This suggests that DL improves the grain indexing.

#### 3.3.2. GB positions   

**Figure 7:**
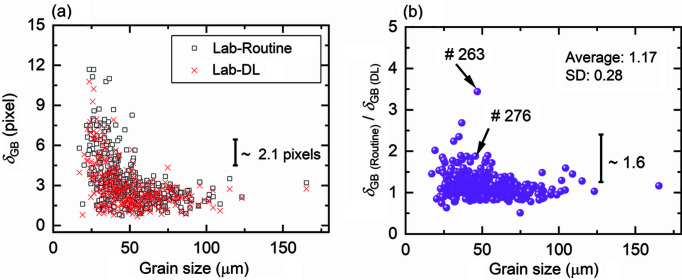
Comparison of accuracies in GB positions between datasets of Lab-Routine and Lab-DL for the common 388 one-to-one indexed grains. (*a*) δ_GB_ and (*b*) δ_GB_ ratios of the standard routine to DL as a function of grain size. Error bars for each data point are not plotted for visual purpose but an average value of the error bar (2.1 and 1.6 pixels, respectively) is plotted instead. In (*b*) the average δ_GB_ ratio is 1.17 and the SD is 0.28. The δ_GB_ ratios for grains #263 and #276 are pointed out by arrows.

There are 388 commonly indexed grains between Lab-Routine and Lab-DL. The boundary positions of these grains in the LabDCT datasets are compared with those in SR-DCT. Fig. 7 ▸(*a*) plots the average deviation of the GB voxels (δ_GB_) as a function of grain size (*D*) for both Lab-Routine and Lab-DL. In both datasets, values of δ_GB_ are low and of similar magnitude for large grains, while they rapidly increase with decreasing grain size for *D* < 40 µm. This is consistent with our previous observation that δ_GB_ is grain size dependent and is larger for smaller grains (Fang *et al.*, 2021 ▸). Comparison for each individual grain shows that δ_GB_ is generally smaller in Lab-DL than in Lab-Routine [Fig. 7 ▸(*a*)], especially for the smaller grains. This becomes more evident when the δ_GB_ ratio of Lab-Routine to Lab-DL is plotted as a function of grain size [Fig. 7 ▸(*b*)]. The figure shows that the ratio is larger than one for 73% of the grains. The average value is 1.17 with a maximum value of 3.4 and the SD is 0.28. This suggests that GB positions are more accurately reconstructed with DL than with the standard routine. As the accuracy of the GB position is an indicator of spatial resolution, it can be concluded that on average the spatial resolution can be improved by 17% using DL instead of the standard routine.

**Figure 8:**
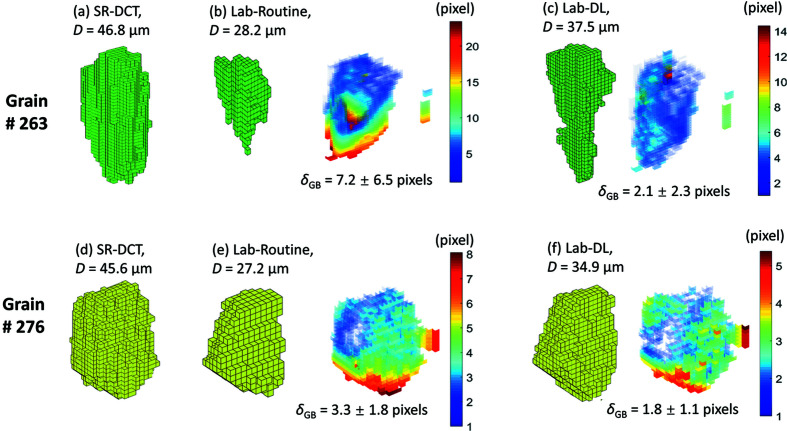
Comparison of GB positions between the two LabDCT datasets (Lab-Routine and Lab-DL) and SR-DCT for (*a*)–(*c*) grain #263 and (*d*)–(*f*) grain #276. For the LabDCT datasets, deviation maps of GB positions are shown in connection with voxelized volumes. Pixels that completely match are shown as transparent in the deviation maps. The values of δ_GB_ are given for each deviation map. The grain size (denoted as *D*) is given for each reconstruction.

Examples of comparing GB deviations for each voxel (ɛ_GB_) are shown for grains #263 and #276 in Figs. 8 ▸(*a*)–8 ▸(*c*) and 8 ▸(*d*)–8 ▸(*f*), respectively. The two grains have a similar grain size of ∼46 µm, and for both grains Lab-DL gives a more precise grain size than Lab-Routine. For grain #263 the values of ɛ_GB_ are as high as 25 pixels for a considerable number of GB voxels in Lab-Routine, whereas they are at highest 14 pixels for only a handful of GB voxels in Lab-DL. As a result, the average value of ɛ_GB_, *i.e. δ*_GB_, for grain #263 in Lab-Routine is 3.4 times that in Lab-DL [see also the marked data point for the ratio in Fig. 7 ▸(*b*)]. For grain #276 the difference is smaller but still observable, showing a smaller δ_GB_ in Lab-DL than Lab-Routine [a δ_GB_ ratio of 1.9, as shown in Fig. 7 ▸(*b*)].

#### 3.3.3. Reasons for better grain indexing and shape reconstruction with DL   

**Figure 9:**
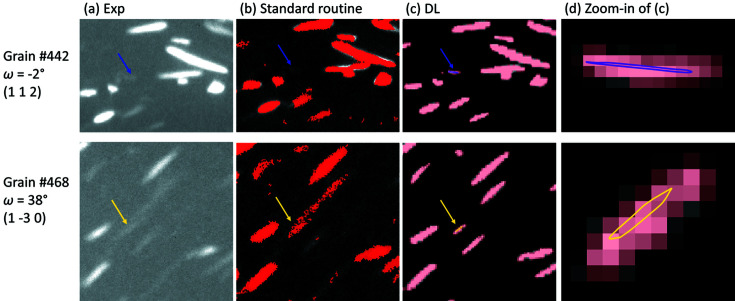
Comparison of spot segmentation using the standard routine and DL for grains at a specific rotation angle (ω). The figure shows (*a*) regions of interest cropped from experimental LabDCT projections, (*b*) segmented images using the standard routine and (*c*) segmented images using DL. Since the two grains are correctly indexed with DL, the forward simulated spots are overlaid onto (*c*) with the zoom-in views shown in (*d*). Arrows mark the location of the spots in each image. The top row shows spot (112) for grain #442 at ω = −2° and the bottom row shows spot 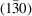 for grain #442 at ω = −38°.

The improved indexing and shape reconstruction (thus spatial resolution) are attributed to a more precise spot segmentation with DL than with the standard routine. As already shown in Fig. 5 ▸, DL has an advantage in segmenting weak spots and also closely located ones, which can be either partly missed or segmented as a single spot with the standard routine. Such difference in spot segmentation can lead to different grain indexing results. As an example, Fig. 9 ▸ shows the differences in spot segmentation as well as indexing results for two grains. For both grains, the experimental spots are rather weak. Thus with the standard routine, they are not segmented for grain #442 or poorly segmented from being connected to a neighboring spot for grain #468 [see Fig. 9 ▸(*b*)]. As a result, neither of them are indexed in Lab-Routine. Conversely, these spots are well segmented with DL [see Fig. 9 ▸(*c*)]. Therefore, the two grains are successfully indexed. This is documented by the fact that forward simulated spots are overlapped with the experimental ones [see Fig. 9 ▸(*d*)].

It can also be seen from Fig. 9 ▸ that the spot boundaries are more precisely identified by DL than by the standard routine for segmenting of the same experimental spots. In general, the spots are segmented as a bit larger/fatter by the standard routine than by DL, due to incorrect noise removal. This is expected to be the main reason for the improved spatial resolution with DL. Some noise is also segmented as small spots with the standard routine [see Fig. 9 ▸(*b*)], which might not be problematic in this case but can cause unexpected false-positive indexing if such incorrect over-segmentation is dominating.

## 4. Discussion   

### 4.1. Advantages of the DL method over the standard routine   

A key to maximizing the performance of the DL method is proper training. As proposed in our preliminary work (Hovad *et al.*, 2020 ▸), this training can be unsupervised (*i.e.* free of tedious and time-consuming human manual labeling) by using forward simulated images. Important here is how to get these forward simulated images to mimic real experimental images. In this work, a procedure combining forward simulated images with the background noise extracted from real experimental images, plus a Poisson noise, is used. It is shown that this procedure makes the input image comprising spatially varying low- and high-frequency noise and the content image ideal for training the DL model. This procedure allows one to create an unlimited number of images. Notably, this method for generating synthetic input images is of a generic nature and can be applied to synthesize images for other DL neural networks.

In the present study, the SNRs in the synthesized input images cover the whole range of typical experimental LabDCT images (from 0.1 to 28, see Fig. S1). It is presumed that the difference in performance between the DL method and the standard routine would be less significant if the SNR was further increased. Much better SNRs would however require longer experimental measuring times. As it is often not realistic to spend such long times, which would also increase source drifts *etc.*, we do not investigate situations with very high SNR values. The main aim of using the DL method is to improve segmentation of small and/or weak spots (usually with low SNR), which are critical for improving grain mapping. A further in-depth investigation to optimize DL performance for LabDCT images with low or ultra low SNRs, resulting from short exposure times, will be of higher value in practice to save measuring time without sacrificing the grain-reconstruction quality.

Mainly two limitations have been recognized for the standard routine: (1) the spot segmentation is sensitive to filtering parameters and thus the parameters must be carefully tuned; and (2) spots, especially the ones with relatively low SNRs, are often under- or over-segmented even with optimized parameter settings. The former limitation requires extensive human expertise, and the latter hinders the improvement of both the detection limit and the spatial resolution for LabDCT. Notably, there is no single set of parameters that work properly for all the spots with the standard routine. There is always a trade-off between saving weak/small spots and removing spots from noise (Lindkvist *et al.*, 2021 ▸). Using the DL method, the background noise can be completely removed and the remaining spots can be segmented easily. During the training of the DL model, a few parameters such as batch size, image size and number of epochs have to be adjusted. However, the basis for the adjustment is simple – just to maximize the values for these parameters within the limits of the capability of the computer system [*e.g.* the graphics processing unit (GPU) memory size].

### 4.2. Versatility of the DL model   

**Figure 10:**
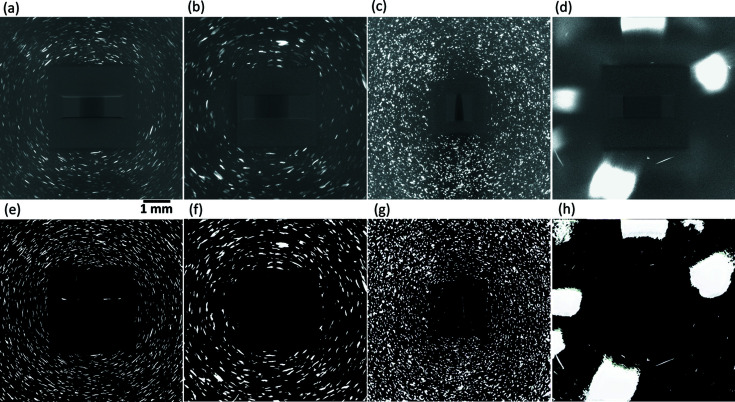
(*a*)–(*d*) Different types of LabDCT images and (*e*)–(*h*) the corresponding DL output images. The sample information and acquisition conditions of the experimental images are as follows: (*a*) a fully recrystallized pure iron with an average grain size of ∼40 µm, an exposure time of 500 s, no pixel binning and *L*_ss_ = *L*_sd_ = 13 mm; (*b*) the same sample, exposure time and pixel binning as (*a*), but *L*_ss_ = 11 mm and *L*_sd_ = 18 mm; (*c*) a fully recrystallized pure iron sample with an average grain size of 25 µm, an exposure time of 300 s, a pixel binning of 2 and *L*_ss_ = *L*_sd_ = 11 mm; and (*d*) a partially recrystallized aluminium sample with an exposure time of 600 s, no pixel binning and *L*_ss_ = *L*_sd_ = 14 mm. The average SNRs for images (*a*)–(*d*) are 3.9 ± 1.9, 4.1 ± 2.0, 4.6 ± 1.4 and 1.4 ± 0.5, respectively.

The trained DL model has been demonstrated to be remarkably efficient in removing background noise, thereby enabling a precise spot segmentation for the LabDCT images from the iron sample in the Laue focusing geometry as shown in Figs. 4 ▸, 5 ▸ and 9 ▸. It also performs rather well for other LabDCT images. This is illustrated in Fig. 10 ▸. It can be seen that all the DL output images for various types of experimental images, measured under different conditions (exposure time, geometries, *etc*.) or for different samples, show a zero background whilst the spots are left brightly visible. The images shown in Figs. 10 ▸(*a*)–10 ▸(*c*) represent the most common types of LabDCT images for fully recrystallized samples with grain sizes in the range 5–200 µm. A very different type of image measured for a partially recrystallized aluminium sample is shown in Fig. 10 ▸(*d*). Also for this image, the DL model performs adequately. This is impressive as no partially recrystallized grain structures were included in the training of the model and the SNR range is slightly lower than those used for training the DL model. All the spots in Fig. 10 ▸(*d*) are recovered as shown in Fig. 10 ▸(*h*), and only the large ‘blobs’, caused by diffraction from a deformed grain in the sample, are overestimated due to streaks (but still with intensities significantly higher than the background). The method for synthesizing images can easily be adapted to include this particular type of LabDCT image for training the DL model if an even more precise spot segmentation is desired.

The efficient noise removal for the different types of images demonstrates the excellent versatility of the DL model. Given the generic nature of the procedure to prepare images for training the DL model, the model is flexible and easy to retrain to include new types of LabDCT images (*e.g.* a very different type of sample or background noise). Therefore, the output can be optimized and a better grain mapping can be obtained. Given its significant advantages over the standard routine and its efficiency for implementation, the DL method offers substantial opportunities to advance the LabDCT technique.

## 5. Conclusions   

We have developed a DL model to clean the background noise of LabDCT images for efficient spot identification. The DL model is trained in an unsupervised manner, avoiding the need for human labeling and intervention. This is realized by combining a forward simulation model and a generic procedure to extract experimental background noise, resulting in input images that are as real as experimental images and content images that are ideal as the ground truth for the training. The following conclusions can be drawn.

(*a*) The DL model is excellent in removing the background noise for various types of LabDCT images, making the model a sophisticated tool to handle challenging circumstances of spot identification, *e.g.* to identify weak or overlapped spots. It is fast and straightforward to apply the DL model to real experimental images once it is trained. The DL method enables more precise spot segmentation in a more straightforward way (no need to tune parameters) compared with the standard routine.

(*b*) The DL model improves grain mapping compared with the results of the standard routine. This was demonstrated by grain reconstructions of an iron sample. Compared with the standard routine, more than ten grains (mostly <30 µm, which are typically challenging to index) were indexed and the spatial resolution (*i.e.* accuracy of the GB position) improved by 17% on average using the DL method.

The proposed approach allows one to create an unlimited amount of training data based on the forward simulations, thereby circumventing the common challenge of having a limited amount of training data. This approach is transferrable and can easily export training data for other DL models with other purposes. The versatility and the other advantages over the standard routine make the DL model a promising method for improving grain mapping techniques.

## Supplementary Material

Supporting information. DOI: 10.1107/S2052252521005480/ro5028sup1.pdf

Source programming code, training dataset, trained model and DL output images: https://doi.org/10.13011/m3-2z1n-qh56

## Figures and Tables

**Figure 11:**
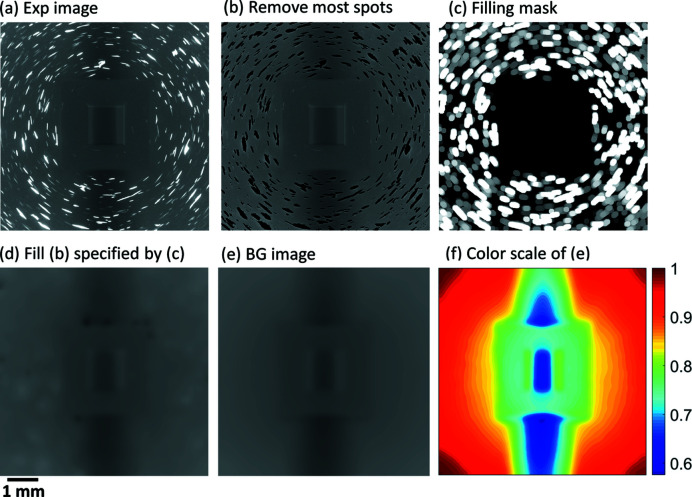
Creating a normalized background-noise image from experimental LabDCT projections. The figure shows (*a*) an experimental LabDCT projection. For part (*b*) most of the spots in the experimental projection are identified and removed by a segmentation method combining a Sobel filtering and binary thresholding. For (*c*) a filling-mask image is obtained by convoluting the image containing the segmented spots with a Gaussian filter (size = 2 pixels and SD = 1 pixel) followed by a dilation in a disk shape with a radius of 13 pixels. Part (*d*) involves filling the regions in (*b*) specified by the mask image (*c*), while (*e*) is the background-noise image obtained by rolling median over 60 sequential images of (*d*) followed by dividing by the maximum pixel value. Finally, (*f*) is a color scale of (*e*) showing the heterogeneous gradients of intensities.

**Table 1:** Characteristics of three virtual input aluminium samples Mean values and standard deviations are given for grain size and the number of spots per projection.

Name	Cylinder dimension, diameter × height (µm^2^)	Number of grains	Grain size (µm)	Number of spots per projection
D18	100 × 150	230	18.3 ± 7.7	391 ± 18
D35	200 × 300	337	35.8 ± 8.2	717 ± 22
D64	400 × 600	485	63.6 ± 14.1	1028 ± 26

**Table 2:** Statistics of indexed grains in the synchrotron dataset (SR-DCT), and in the LabDCT datasets obtained by the standard routine (Lab-Routine) and deep learning (Lab-DL) Grain size is expressed as a mean value, 〈*D*〉, with a standard deviation. For one-to-multi indexed grains, the number of grains, *N*, in SR-DCT is given as well as the paired number of grains in the LabDCT datasets listed in brackets.

Dataset	〈*D* 〉 (µm)	*N*				
		Total indexed	One-to-one indexed	One-to-multi indexed	False-negatively indexed	False-positively indexed
SR-DCT	39.6 ± 22.4	596	—	—	—	—
Lab-Routine	42.9 ± 27.0	418	403	5 (10)	188	5
Lab-DL	43.6 ± 26.0	429	415	4 (8)	177	6

## Appendix A Generation of a normalized background-noise image

As shown in Fig. 11 ▸, spots in an experimental image are segmented by a combination of Sobel filtering (Sobel, 2014 ▸) and binary thresholding; this segmentation does not need to be ideal as the purpose is only to remove most of the bright spots from the input, as shown in Fig. 11 ▸(*b*). Then, a filling-mask image [Fig. 11 ▸(*c*)] is generated to define the regions with nonzero pixel values for filling the neighboring ‘holes’, followed by a Gaussian filtering, resulting in Fig. 11 ▸(*d*). To get rid of the local intensity variations caused by the filling process, a rolling median process over a large number (*e.g.* 60) of sequential images is applied. The obtained image is subsequently normalized by dividing all pixel values by the maximum one, rendering the final image as shown in Fig. 11 ▸(*e*). Fig. 11 ▸(*f*) shows the final image in a pseudo-colored version.
