# Supplementary material for: Deep learning for improving non-destructive grain mapping in 3D
Source: IUCrJ. 2021 Jul 15;8(Pt 5):719–31. doi: 10.1107/S2052252521005480 (PMC8420763; doi:10.1107/S2052252521005480)
Supplement: Supplementary file 1 [file m-08-00719-sup1.pdf]

IUCrJ

Volume 8 (2021)

Supporting information for article:

Deep learning for improving non-destructive grain mapping in 3D

H. Fang, E. Hovad, Y. Zhang, L. K. H. Clemmensen, B. Kjaer Ersbøll and D. Juul Jensen

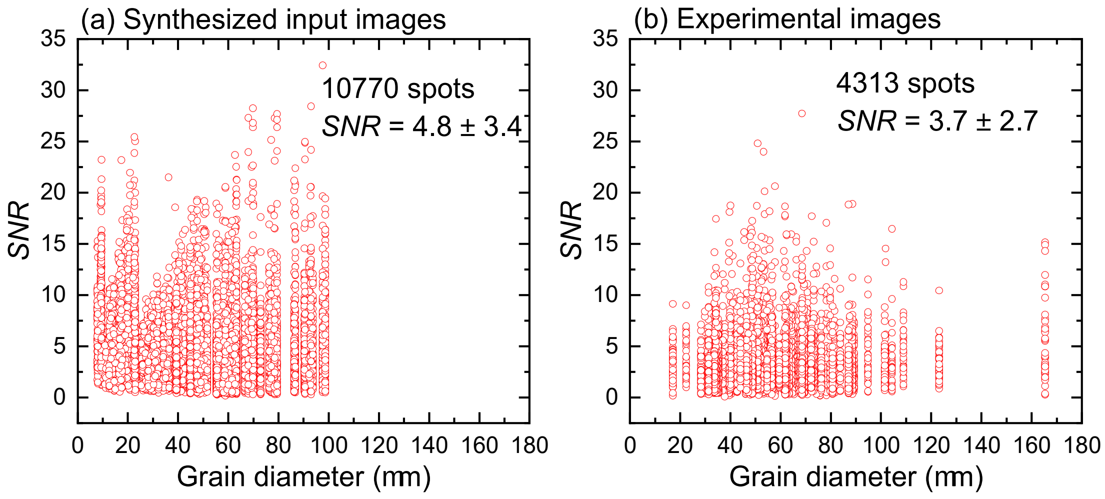

Figure S1 Signal-to-noise ratio (*SNR*) for randomly sampled spots as a function of grain diameter in (a) synthesized DL input images and (b) experimental images for the iron sample measured by LabDCT as presented in the main text. The *SNR* is defined as $SNR = (\bar{I}_{spot} - \bar{I}_{bg})/\sigma_{bg}$, where $\bar{I}_{spot}$ is average spot intensity, $\bar{I}_{bg}$ is average local background intensity and σ_{bg} is standard deviation of the local background intensity distribution in the spot bounding box excluding other spots. In (a) *SNR* has an average value of 4.8 and a standard deviation of 3.4. In (b) *SNR* has an average value of 3.7 and a standard deviation of 2.7.

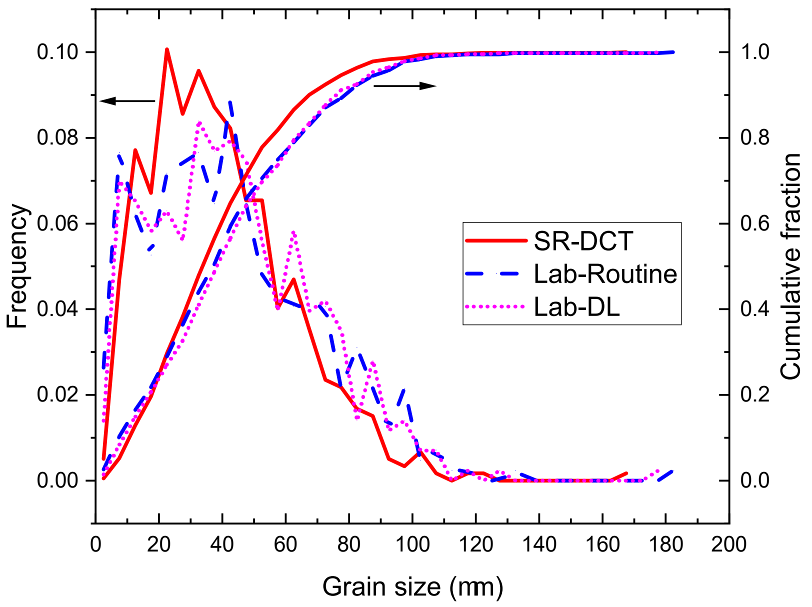

Figure S2 Size distribution of reconstructed grains in the three datasets: synchrotron DCT dataset (SR-DCT) and LabDCT datasets with the standard routine (Lab-Routine) and the DL method (Lab-DL).

Table S1 Summary of the 54 convolutional layers in the dynamic U-Net network. The output shape is expressed by the number of feature channels, image width and height. The number of parameters is also given for each operation together with an indication of whether the corresponding parameters are trainable or not. Layer 1 - 39 is for downsampling and layer 40 - 54 is for upsampling.

Convolutional layer number	Layer (type)	Output shape	Parameter #	Trainable
1	Conv2d	[64, 300, 300]	9,408	False
	BatchNorm2d	[64, 300, 300]	128	True
	ReLU	[64, 300, 300]	0	False
	MaxPool2d	[64, 150, 150]	0	False
2 – 7	Conv2d	[64, 150, 150]	36,864	False
	BatchNorm2d	[64, 150, 150]	128	True
	ReLU	[64, 150, 150]	0	False
8 - 16	Conv2d	[128, 75, 75]	147,456	False
	BatchNorm2d	[128, 75, 75]	256	True
	ReLU	[128, 75, 75]	0	False
17 - 29	Conv2d	[256, 38, 38]	589,824	False
	BatchNorm2d	[256, 38, 38]	512	True
	ReLU	[256, 38, 38]	0	False
30 - 36	Conv2d	[512, 19, 19]	2,359,296	False
	BatchNorm2d	[512, 19, 19]	1024	True
	ReLU	[512, 19, 19]	0	False
37	Conv2d	[1024, 19, 19]	4,719,616	True
	ReLU	[1024, 19, 19]	0	False
38	Conv2d	[512, 19, 19]	4,719,104	True
	ReLU	[512, 19, 19]	0	False
39	Conv2d	[1024, 19, 19]	525,312	True
	PixelShuffle	[256, 38, 38]	0	False
	ReplicationPad2d	[256, 39, 39]	0	False

	AvgPool2d	[256, 38, 38]	0	False
	ReLU	[1024, 19, 19]	0	False
	BatchNorm2d	[256, 38, 38]	512	True
40	Conv2d	[512, 38, 38]	2,359,808	True
	ReLU	[512, 38, 38]	0	False
41	Conv2d	[512, 38, 38]	2,359,808	True
	ReLU	[512, 38, 38]	0	False
	ReLU	[512, 38, 38]	0	False
42	Conv2d	[1024, 38, 38]	525,312	True
	PixelShuffle	[256, 76, 76]	0	False
	ReplicationPad2d	[256, 77, 77]	0	False
	AvgPool2d	[256, 76, 76]	0	False
	ReLU	[1024, 38, 38]	0	False
	BatchNorm2d	[128, 75, 75]	256	True
43	Conv2d	[384, 75, 75]	1,327,488	True
	ReLU	[384, 75, 75]	0	False
44	Conv2d	[384, 75, 75]	1,327,488	True
	ReLU	[384, 75, 75]	0	False
	ReLU	[384, 75, 75]	0	False
45	Conv2d	[768, 75, 75]	295,680	True
	PixelShuffle	[192, 150, 150]	0	False
	ReplicationPad2d	[192, 151, 151]	0	False
	AvgPool2d	[192, 150, 150]	0	False
	ReLU	[768, 75, 75]	0	False
	BatchNorm2d	[64, 150, 150]	128	True
46	Conv2d	[256, 150, 150]	590,080	True

	ReLU	[256, 150, 150]	0	False
47	Conv2d	[256, 150, 150]	590,080	True
	ReLU	[256, 150, 150]	0	False
	ReLU	[256, 150, 150]	0	False
48	Conv2d	[512, 150, 150]	131,584	True
	PixelShuffle	[128, 300, 300]	0	False
	ReplicationPad2d	[128, 301, 301]	0	False
	AvgPool2d	[128, 300, 300]	0	False
	ReLU	[512, 150, 150]	0	False
	BatchNorm2d	[64, 300, 300]	128	True
49	Conv2d	[96, 300, 300]	165,984	True

49	ReLU	[96, 300, 300]	0	False
50	Conv2d	[96, 300, 300]	83,040	True
	ReLU	[96, 300, 300]	0	False
	ReLU	[192, 300, 300]	0	False
51	Conv2d	[384, 300, 300]	37,248	True
	PixelShuffle	[96, 600, 600]	0	False
	ReLU	[384, 300, 300]	0	False
	MergeLayer	[99, 600, 600]	0	False
52	Conv2d	[99, 600, 600]	88,308	True
	ReLU	[99, 600, 600]	0	False
53	Conv2d	[99, 600, 600]	88,308	True
	ReLU	[99, 600, 600]	0	False
	MergeLayer	[99, 600, 600]	0	False
54	Conv2d	[3, 600, 600]	300	True

Table S2 Total volume of indexed grains in the three different datasets.

Dataset	Total volume of indexed grains, ($\times 10^7$ μm^3)	Relative difference compared to SR-DCT
SR-DCT	4.1401	-
Lab-Routine	4.1371	0.07%
Lab-DL	4.1349	0.13%
